# Supplementary material for: Metabolomic Study of Dactylis glomerata Growing on Aeolian Archipelago (Italy)
Source: Metabolites. 2022 Jun 9;12(6):533. doi: 10.3390/metabo12060533 (PMC9229457; doi:10.3390/metabo12060533)
Supplement: Supplementary file 1 [file metabolites-12-00533-s001.zip › metabolites-1761546-supplementary.pdf]

Table S1. Semi-quantitative analysis by ^{1}H NMR of the metabolites detected in *D. glomerata* growing on Stromboli island. Values are expressed as μg of metabolite / mg of dried plant. Each value is a mean of 5 values (for each altitude 5 individuals were collected). Chemical shift and number of protons used for the semi-quantification are reported. A color scale from red to green for each metabolite indicates growing amount of that metabolite whithin different samples.

Metabolite	NMR signal used for the semi-quantification	100 m a.s.l.	200 m a.s.l.	400 m a.s.l.	500 m a.s.l.	700 m a.s.l.
alanine	δ 1.49, 3H	0.958	2.01	2.07	1.634	1.498
glucose*	δ 5.2, 1H<br>δ 4.6, 1H	15.376	14.152	10.08	15.928	5.816
malic acid	δ 2.72, 1H	8.95	15.014	20.68	21.632	21.652
sucrose	δ 5.4, 1H	38.696	47.31	53.356	60.89	22.034
fumaric acid	δ 6.54, 2H	0.04	0.052	0.11	0.098	0.18
asparagine	δ 2.95, 1H	0.214	1.016	1.792	1.124	9.22
chlorogenic acid	δ 6.37, 1H	5.356	4.498	4.17	2.934	2.382
acetic acid	δ 1.96, 3H	0.512	0.746	0.798	0.708	0.81
phenylalanine	δ 7.34, 5H	0.494	0.894	1.094	0.612	1.314
proline	δ 2.04, 4H	5.852	12.442	11.842	8.184	7.826
threonine	δ 1.34, 3H	1.354	1.494	1.684	1.42	2.394
valine	δ 1.06, 3H	0.768	1.648	2.2	1.21	2.028
glycine betaine	δ 3.28, 9H	9.054	6.488	6.448	8.16	6.878

*Glucose amount is calculated as sum of α-glucose (δ 5.2) and β-glucose (δ 4.6).

Table S2. Coordinates of the sampling sites.

Station	Longitude	Latitude
Vul1	14.948277	38.401176
Vul2	14.949785	38.394727
Vul3	14.958942	38.39065
Lip1	14.906545	38.47771
Lip2	14.912074	38.477547
Lip3	14.926652	38.484155
Lip4	14.932741	38.485265
Pan1	15.055597	38.629839
Pan2	15.059322	38.632535
Pan3	15.062572	38.636458
Str1	15.232169	38.800831
Str2	15.227405	38.802299
Str3	15.224399	38.798646
Str4	15.222381	38.797452
Str5	15.218622	38.794341

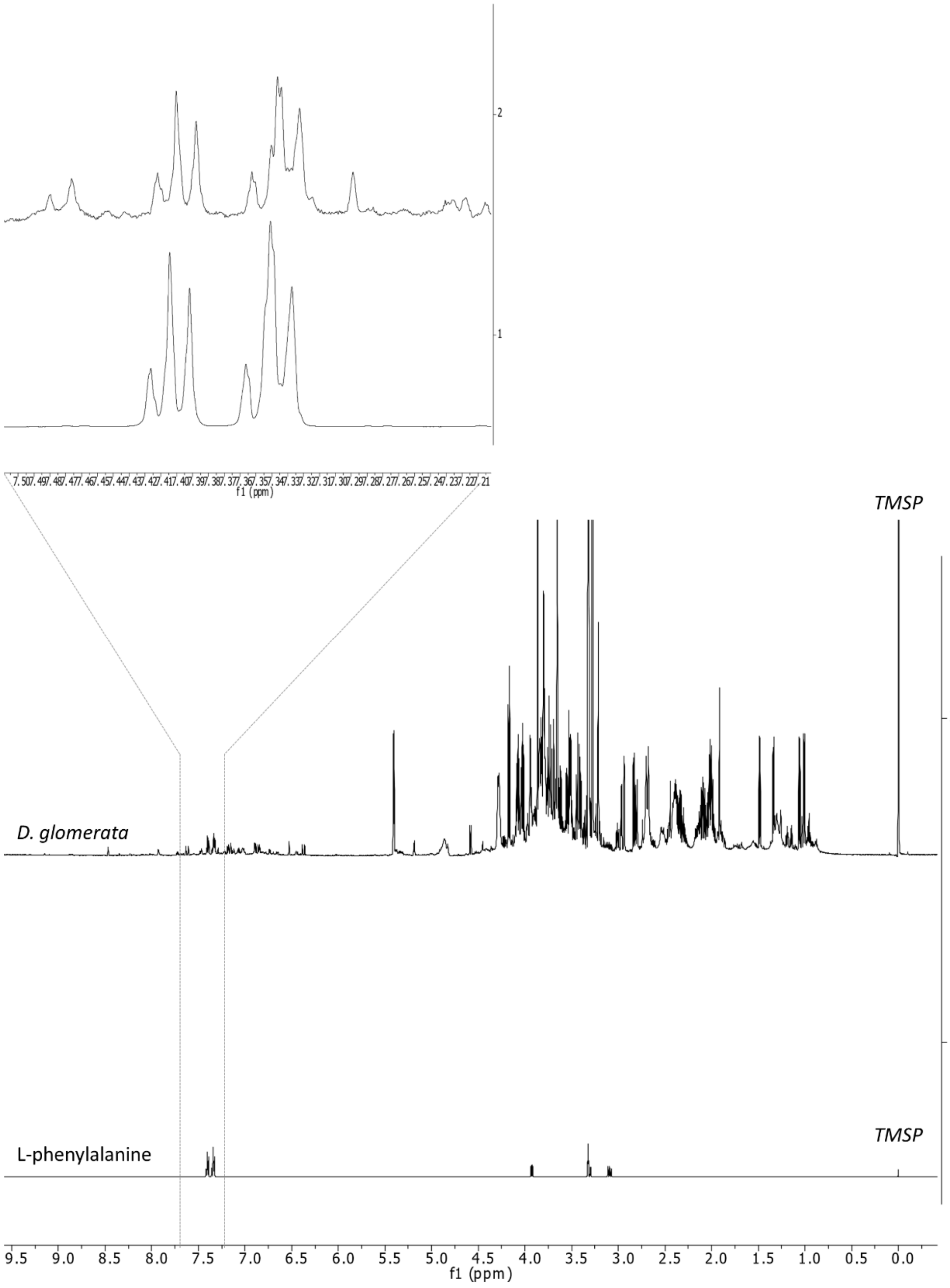

Figure S1. Comparison between ^{1}H NMR spectrum of *D. glomerata* extract (plant growing at 700 m a.s.l.) and L-phenylalanine standard. The enlarged region between δ 7.5 – 7.2 shows the diagnostic signals of the aromatic protons.

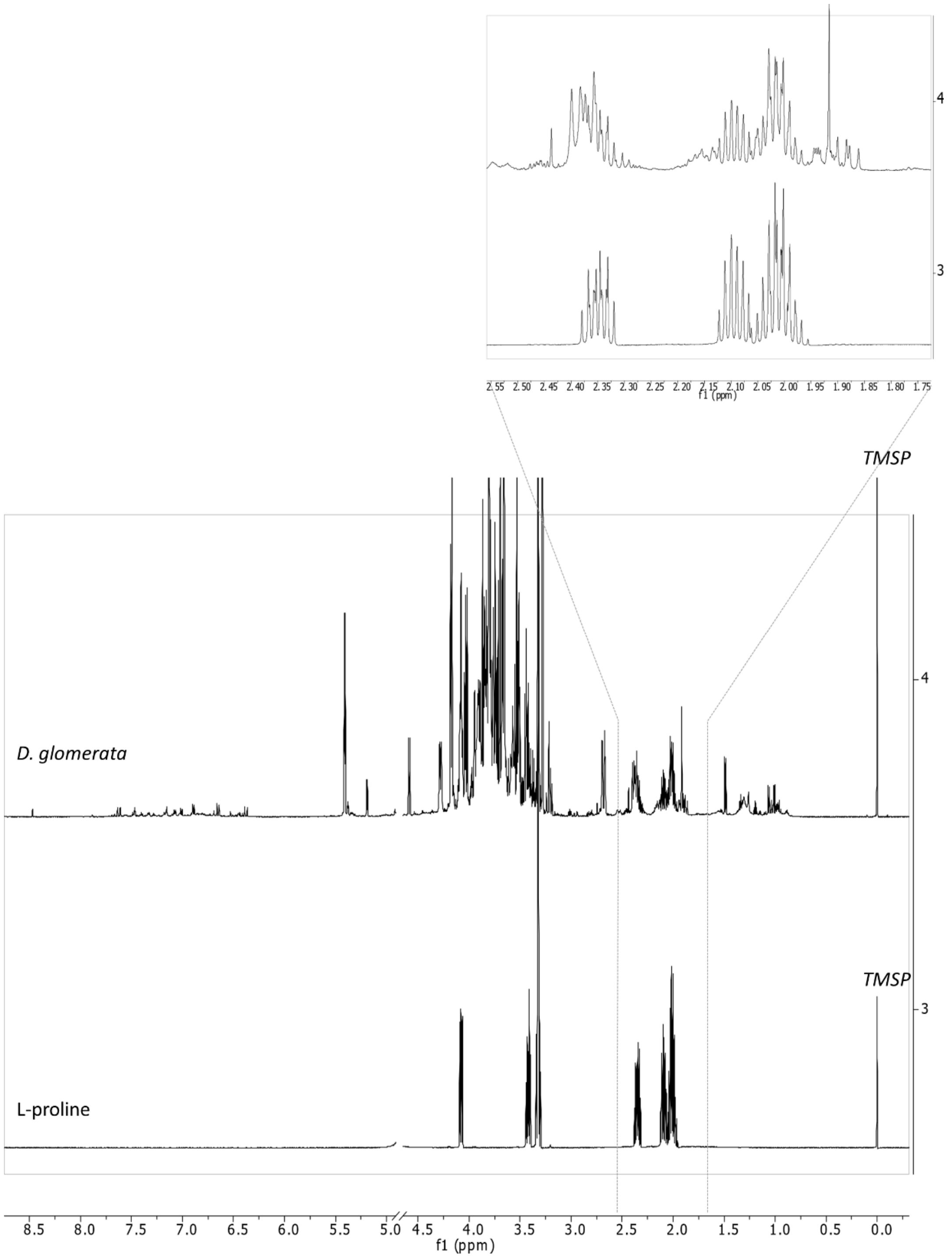

Figure S2. Comparison between ^{1}H NMR spectrum of *D. glomerata* extract (sample growing at 500 m a.s.l.) and L-proline standard. The enlarged region between δ 2.5 – 1.7 shows the diagnostic signals of the aromatic protons.

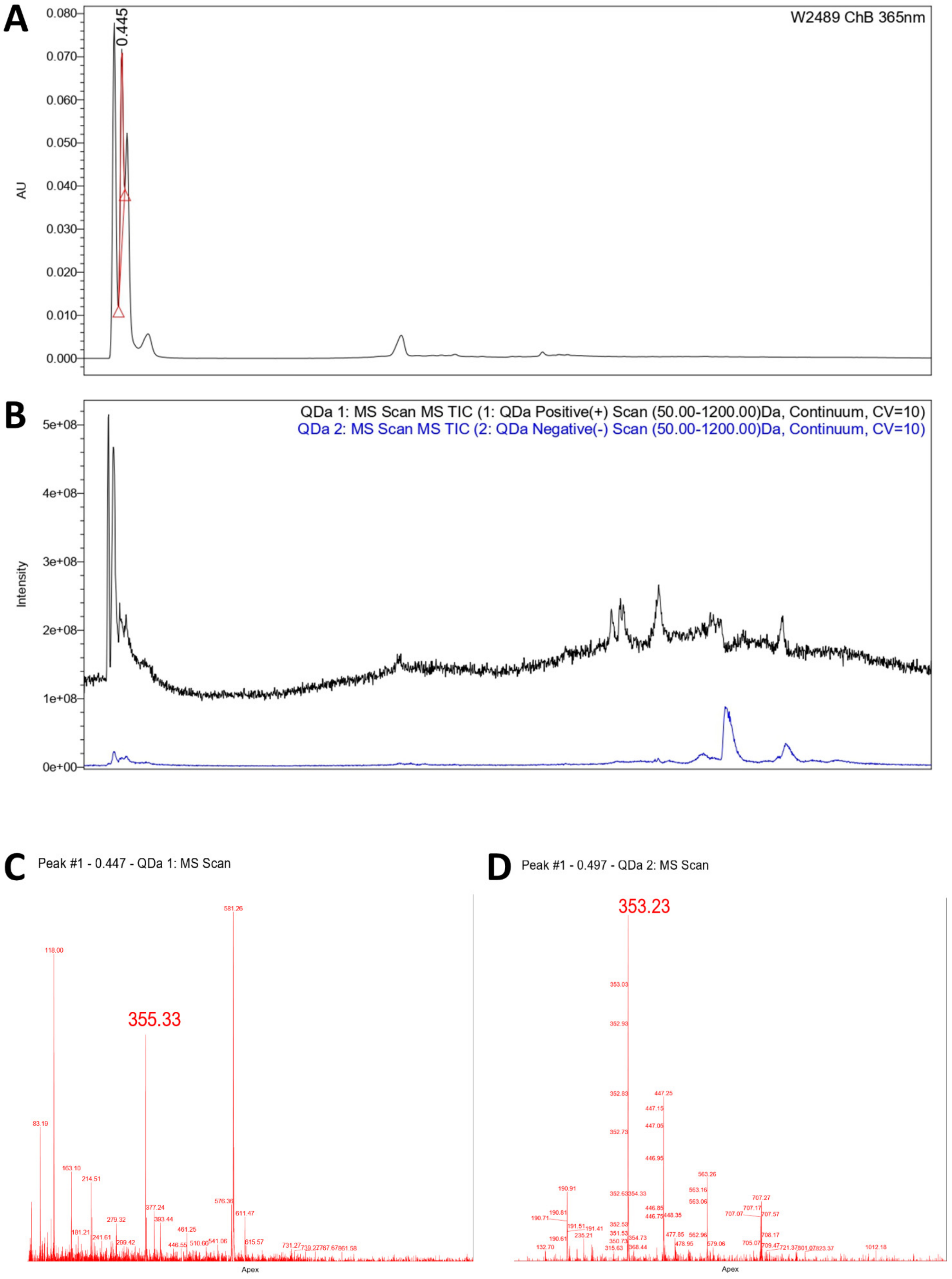

Figure S3. UHPLC–MS analysis of *D. glomerata* sample growing on Stromboli at 100 m a.s.l. **A**) UV chromatogram at λ 365 nm. **B**) Total ion chromatograms in positive ion mode -QDa 1- (black trace) and negative ion mode-QDa 2- (blue trace). Peak highlighted in red in (**A**) at retention time 0.445 min was interpreted as cholorogenic acid (exact mass 354.095 Da) which gave m/z value 355.33 [M+H] in QDa 1 (**C**) and 353.23 [M-H] in QDa 2 (**D**).
